# Research on Body Composition and Lifestyle Behaviors During Pubertal Development in 6–12-Year-Old Children with Obesity

**DOI:** 10.3390/healthcare13060607

**Published:** 2025-03-11

**Authors:** Jin Zhang, Tian Zhang, Naijun Wan

**Affiliations:** Department of Pediatrics, Beijing Jishuitan Hospital, Capital Medical University, Beijing 100035, China; zjdct@163.com (J.Z.); tianbing1214@sina.com (T.Z.)

**Keywords:** body composition, children, obesity, puberty

## Abstract

**Objective:** The objective of this study was to investigate the pubertal development in school-age children with obesity and to explore the body composition and lifestyle behaviors influencing its onset. **Method:** We enrolled 217 children, aged 6–12 years, who visited the Pediatrics Department at Beijing Jishuitan Hospital, Capital Medical University. All participants underwent a series of examinations. These assessments included body composition analysis, measurement of blood glucose and fasting insulin levels, and evaluation of secondary sexual characteristics. Statistical analysis was conducted using R4.0.3 software. **Results:** Of the 152 male participants, 83 (54.6%) were in the adolescent-undeveloped group, and 69 (45.4%) were in the adolescent-developed group. Of the 119 female students, 30 (25.2%) were in the adolescent-undeveloped group, and 89 (74.8%) were in the adolescent-developed group. In a comparative analysis of children, those in the adolescent development group exhibited significantly higher values for age, height, weight, body mass index (BMI), BMI-z score, body fat, muscle mass, fat-free weight, fat-free body mass index, and waist/hip ratio compared to the adolescent-undeveloped group (*p* < 0.05). Additionally, fasting insulin and insulin resistance index were also higher in the development group, with statistical significance observed. BMI emerged as an independent factor affecting the adolescent development of school-age girls with obesity (*p* < 0.05). Among boys in the adolescent development group, the consumption of sugary drinks and fried food was higher than in the undeveloped group, and moderate-intensity exercise was significantly lower, with statistical significance (*p* < 0.05). In girls, the adolescent development group reported longer daily sitting times and higher intakes of sugary drinks and fried foods compared to the undeveloped group, with these differences being statistically significant (*p* < 0.05). The consumption of fried food was positively correlated with adolescent development in children with obesity and was identified as an independent influencing factor of adolescent development (*p* < 0.05). **Conclusions:** A high body mass index (BMI) in girls with obesity and high intake of fried foods in both genders are strong predictors of early puberty in school-age children.

## 1. Introduction

Obesity among children and adolescents has emerged as a paramount issue in global health. Over the past 40 years, there has been a marked increase in the prevalence of obesity among children of all ages globally [1]. The disparity between high caloric intake and low energy expenditure results in a surplus of energy, which leads to the accumulation of adipose tissue. The etiology of obesity is complex and involves multiple factors, including nutrition, development, behavior, genetics, environment, epigenetics, and prenatal influences. Childhood obesity not only elevates the long-term risk of cardiovascular diseases, dyslipidemia, obstructive sleep apnea, certain cancers, and premature mortality but also poses detrimental effects on the reproductive system. These effects can manifest as precocious puberty, irregular menstrual cycles, polycystic ovary syndrome, and an increased propensity for high-risk sexual behaviors [2]. An increasing body of research indicates that children with obesity tend to initiate puberty at an earlier age compared to their normal-weight peers [3]. Obesity and early-onset puberty are significant risk factors for the development of metabolic syndrome, type 2 diabetes, and insulin resistance [4]. Puberty development is influenced by a multitude of factors, including heredity, nutrition, environment, and lifestyle behaviors. Among these, metabolic conditions and energy reserves are pivotal in regulating the onset of puberty. There is robust evidence supporting the impact of higher body mass index (BMI) on both the initiation and progression of puberty in both boys and girls. The age at which girls experience breast development is progressively earlier and is positively correlated with BMI. Additionally, boys with obesity of the same age are more likely to undergo sexual maturation than their counterparts without obesity [5,6,7]. Some studies find that obesity accelerates puberty in boys, others find the opposite. Previous studies have indicated a negative linear correlation between BMI and the onset of puberty in males, suggesting that individuals with early puberty development tend to have a lower percentage of body fat [8,9]. In addition to body composition factors such as weight and fat tissue, dietary habits, physical activity, and sleep patterns of children with obesity also subtly influence the process of puberty development. However, the factors influencing the pubertal development of children with obesity and the underlying mechanisms remain inconclusive. To address this gap, we conducted a study observing the body composition, sexual maturation, and lifestyle behaviors of 271 children aged 6–12 with obesity. The metabolic indicators of children with obesity, such as blood glucose and insulin, were also monitored simultaneously. We analyzed potential influencing factors on the pubertal development of these children and investigated the possible mechanisms by which body fat might participate in the activation of the gonadal axis. The findings of this research are presented below.

## 2. Materials and Methods

### 2.1. Research Objects and Groups

The study subjects were children with obesity aged 6–12 who underwent physical examinations at the Pediatrics Department of Beijing Jishuitan Hospital, Capital Medical University. We posted recruitment advertisements in the pediatric clinic area to ensure the continuity of enrolled volunteers and established a professional fast-track for the enrollment medical examination. The enrollment period was from October 2018 to December 2023. The diagnostic criteria for childhood obesity were based on the body mass index (BMI) cut-off points, BMI ≥ 95th percentile, according to the Chinese criteria for obesity recommended by the Expert Group on ‘Evaluation’, Treatment, and Prevention of Childhood Obesity in China’ [10]. Exclusion criteria included ① children with liver or kidney dysfunction; ② children with endocrine or metabolic disorders, autoimmune diseases, or other conditions that could impact bone metabolism; ③ a history of drug poisoning; and ④ secondary obesity resulting from other factors. A total of 271 subjects met the inclusion criteria. The criteria for the assessment of pubertal onset were as follows: girls with breast development at or above Tanner II stage and boys with testicular volume of 4 mL or greater. They were considered to have initiated pubertal development [11]. Children with obesity were categorized into two groups: the puberty development group and the puberty undeveloped group, based on the presence or absence of adolescent development. The study protocol was approved by the Institutional Review Board of Beijing Jishuitan Hospital, Capital Medical University (No.201808-03). Informed consent was obtained from all participants and their legal guardians prior to enrollment.

### 2.2. Methods

#### 2.2.1. Physical Examination and Body Composition Assessment

The physical examination, body composition, and bone density measurements for all subjects were independently conducted by the same surveyor, with quality control measures implemented each time to calibrate the precision and accuracy of the instruments. Subjects were dressed in light clothing, and their height (in centimeters) and weight (in kilograms) were measured, with the final value being the average of two measurements. The body mass index (BMI) was calculated as weight (in kilograms) divided by the square of height (in meters). Bioelectrical impedance analysis (BIA) was employed to measure body composition with a Sihai Huachen H-Key350 eight-electrode BIA detector (Shanxi Si Hai Hua Chen Technology Co., Ltd., Xi’an, China). The subjects emptied their bladders (and did not eat or drink water for 2.5 to 3 h before measurement), wore light clothing, removed all metal objects and accessories, stood barefoot on the measurement panel, held the electrode handle with both hands at the same time, kept silence, and were measured after full electrode contact (contact with both hands and feet). The results for body fat, visceral fat, muscle mass, body fat percentage, and the waist/hip ratio were recorded. The body fat index was defined as body fat (in kilograms) divided by the square of height (in meters). The muscle mass index was defined as muscle mass (in kilograms) divided by the square of height (in meters). The fat-free body mass index was defined as fat-free weight (in kilograms) divided by the square of height (in meters). The Z-score was calculated as (observed value − mean of the value)/standard deviation, which means the difference between the observed value of each subject and the mean of that value, divided by the standard deviation of the value. The physical examination was performed by a pediatric endocrinologist who had undergone uniform training. This examination was designed to assess pubertal development, which included the evaluation of female breast development and male testicular volume. Breast development was assessed through visual examination and palpation, following the Tanner staging criteria. Testicular volume was determined by palpation and comparison with a Prader testicular meter. In cases where there was a difference in testicular size between the two sides, the larger value was recorded. If the stages of breast development on both sides were different, the more mature side was documented.

#### 2.2.2. Determination of Fasting Insulin and Fasting Blood Glucose

After a 12 h fast, 5 mL of venous blood was collected and sent to the Laboratory of Beijing Jishuitan Hospital, Capital Medical University, for testing. Fasting insulin (FIN) was measured using the chemiluminescence method, while blood glucose was detected via the glucose oxidase method. A Hitachi 7600 automatic biochemical analyzer was used for these measurements. The Homeostasis Model Assessment of Insulin Resistance (HOMA-IR) was calculated using the formula HOMA-IR = (fasting blood glucose [mmol/L] × fasting insulin [uU/mL]) /22.5, and the cutoff value for HOMA-IR was greater than 2.5.

#### 2.2.3. Questionnaire Survey Administered by a Professional Pediatrician

The survey questionnaire for this study was determined based on the predictive survey questionnaire and the revision suggestions from pediatric endocrinology experts at our hospital. Pediatric endocrinologists guided the parents (father or mother) of the enrolled volunteers in completing the survey questionnaire to ensure the authenticity and scientific validity of the data. Once the questionnaires were collected, they were entered into a database by two individuals to ensure consistency and logical accuracy. The questionnaire covered a range of topics, including general population characteristics such as date of birth, gender, and birth weight, as well as lifestyle and dietary factors. These factors included nightly sleep duration, engagement in moderate-intensity physical activity, daily sitting time, postnatal feeding methods, and dietary habits such as meat consumption, intake of sugary beverages, and consumption of fried foods in the past year.

#### 2.2.4. Relevant Indicators and Definitions of Lifestyle Behaviors and Dietary Intake

The following definitions were used to categorize the subjects’ lifestyle behaviors and dietary intake: ① Physical Activity Level. More Exercise: Defined as engaging in moderate-intensity exercise for a total of 180 min or more per week. Less Exercise: Defined as engaging in moderate-intensity exercise for less than 180 min per week. ② Meat Consumption. More Meat Intake: Defined as consuming meat 5 times or more per week. Less Meat Intake: Defined as consuming meat less than 5 times per week. ③ Sugary Drink Consumption. More Sugary Drink Intake: Defined as consuming sugary drinks 3 times or more per week. Less Sugary Drink Intake: Defined as consuming sugary drinks less than 3 times per week. ④ Fried Food Consumption. More Fried Food Intake: Defined as consuming fried foods 3 times or more per week. Less Fried Food Intake: Defined as consuming fried foods less than 3 times per week. These definitions were used to classify the subjects’ behaviors and dietary habits for the purpose of the study.

### 2.3. Statistical Analysis

For the statistical analysis, version 4.0.3 of the statistical software was utilized. The Shapiro–Wilk normality test was applied to assess the normality of the sample data for continuous variables. If the data conformed to a normal distribution, the mean and standard deviation were used to represent the data, and the independent samples T-test was employed to compare between two groups. In cases where the data did not conform to a normal distribution, the median along with the 25th and 75th percentiles were used, and the Wilcoxon rank-sum test was applied for group comparisons. Categorical variables were described using frequency counts, and group comparisons were made using the Chi-square test or Fisher’s exact test, depending on the data distribution and sample size. The method of backward selection for independent variables was conducted using the stepwise AIC (Akaike Information Criterion) function. Because the puberty development variable is a categorical variable, for binary logistic regression analysis, the glm function was employed. When the independent variable was a categorical variable, the reference group was the group with the minimum value. When the independent variable was continuous, it was directly included in the binary logistic regression model. Variables with statistically significant differences between groups were included in the logistic regression model for analysis. A difference was considered statistically significant if the *p*-value was less than 0.05.

## 3. Result

### 3.1. Relationship Between Puberty Development and Body Composition and Insulin Resistance in Children with Obesity

This study included a total of 217 participants. There were 152 male students; the average age was 9.58 ± 1.32 years, and the average BMI was 25.6 ± 3.79, with 83 (54.6%) in the adolescent-undeveloped group and 69 (45.4%) in the adolescent-developed group. There were 119 female students; the average age was 8.19 ± 1.44 years, and the average BMI was 22.9 ± 2.71, with 30 (25.2%) in the adolescent-undeveloped group and 89 (74.8%) in the adolescent-developed group. Among the male students, age, height, weight, BMI, BMI z-score, body fat, muscle mass, muscle mass index, fat-free weight, fat-free body mass index, waist/hip ratio, fasting insulin, and insulin resistance index were all higher in the adolescent-developed group compared to the adolescent-undeveloped group. These differences were statistically significant (*p* < 0.05). Among the female students, age, height, weight, BMI, BMI z-score, body fat, muscle mass, fat-free weight, fat-free body mass index, waist/hip ratio, fasting insulin, fasting blood glucose, and insulin resistance index were all higher in the adolescent-developed group compared to the adolescent-undeveloped group. These differences were also statistically significant (*p* < 0.05). These results are detailed in Table 1. After excluding the collinearity factor and adjusting for the confounding effects of age and height, BMI was identified as an independent influencing factor of adolescent development among girls (*p* < 0.05), as shown in Table 2.

### 3.2. The Impact of Lifestyle, Diet, and Other Factors on the Development of Adolescents with Obesity in School-Age Children

In Table 3, it can be observed that the birth weight of boys in the adolescent development group was significantly higher than that of boys in the adolescent-undeveloped group (*p* < 0.05). Additionally, the consumption of sugary drinks and fried foods was notably higher in the adolescent development group compared to the adolescent-undeveloped group (*p* < 0.05). Conversely, the engagement in moderate-intensity exercise was significantly lower in the adolescent development group than in the adolescent-undeveloped group (*p* < 0.05). For adolescent girls, the developmental group exhibited a higher daily sitting time and a greater intake of sugary drinks and fried foods compared to the undeveloped group, with these differences being statistically significant (*p* < 0.05). Multiple regression analysis indicates a positive correlation between the intake of fried foods and the puberty development of both boys and girls, establishing it as an independent influencing factor of puberty development (*p* < 0.05) (Table 4 and Table 5).

## 4. Discussion

Previous research has demonstrated that fat is a vital component for sustaining life and metabolic processes. However, an excess or abnormal accumulation of fat can be detrimental to human health. Obesity is associated not only with an increased risk of various metabolic complications but also impacts adolescent development. In 1993, Frisch, after analyzing the long-term follow-up data of 181 normal children, introduced the concept of “critical weight.” This concept suggests that women require a certain amount of fat reserves to initiate puberty, and body fat is also an essential element for maintaining fertility [12]. Numerous population-based studies have identified a correlation between high BMI in girls and their adolescent development. Our study’s findings indicate that among school-age girls with obesity, those in the adolescent development group exhibit higher weight, BMI, and BMI z-scores compared to those in the adolescent-undeveloped group. BMI is an independent influencing factor in the adolescent development of school-age girls with obesity. Previous research has also substantiated the link between obesity and female puberty development. A meta-analysis of 30 studies reveals that from 1977 to 2013, the age of female breast development has been occurring three months earlier every decade, a trend that coincides with the increasing prevalence of obesity year by year [13]. A cross-sectional study of girls in China indicates that those with overweight or obesity tend to experience earlier development of breasts and pubic hair compared to their peers with a healthy weight [14,15].

The critical BMI value is highly specific for diagnosing obesity, meaning it can accurately identify those who have obesity. However, it has low sensitivity for detecting obesity, which means it may miss some individuals who actually have obesity. BMI does not provide information about the proportions of various components of body weight, such as body fat, muscle mass, fat-free weight, and body fat distribution [16]. BMI, body fat, and muscle mass undergo changes as adolescents develop, and these changes are accompanied by gender-specific differences. Our research has shown that among boys with obesity in the adolescent development group, there are higher levels of body fat, muscle mass index, fat-free body mass index, and waist/hip ratio compared to those in the adolescent-undeveloped group. In contrast, for girls with obesity, we observed higher levels of body fat, fat-free body mass index, and waist/hip ratio. Xu et al. [17] posited that there are discernible gender differences in BMI, waist circumference, and body fat percentage as puberty progresses. Specifically, the body fat percentage in girls tends to increase with the onset of puberty, a pattern that does not hold true for boys. The fat accumulation in girls may largely account for the post-puberty increase in BMI. In contrast, the rise in BMI among boys is more likely due to the accumulation of fat-free mass. An additional study conducted with children with obesity in Beijing, China, demonstrated that female BMI and body fat percentage were positively correlated with early puberty development. In the case of boys, the early puberty development group had a higher BMI but a lower body fat percentage [9]. Dietary habits, cultural differences, and climatic environments in different regions may account for the variations in the correlation between BMI and pubertal development among male children with obesity of different races.

Currently, the mechanism by which obesity affects adolescent development remains unclear. Obesity is associated with a range of changes in metabolic cytokines and hormones, including leptin, gastrin, insulin, and certain central lipids. The alterations in these metabolites may impact the Hypothalamus–Pituitary–Gonad (HPG) axis and play a role in the regulation of adolescent development. Hypothalamic Kisspeptin neurons are thought to be the key nexus between metabolism and adolescent development. Kisspeptin, secreted by the *kiss1* gene, directly acts on gonadotropin-releasing hormone (GnRH) neurons, thereby activating the onset of puberty. The increase in adipose tissue in children with obesity stimulates an increase in leptin secretion, which in turn continues to stimulate the expression of the *kiss1* gene. This process may accelerate the onset of puberty. Leptin and *Kiss1* neurons are interconnected in central brain regions and may overlap, suggesting that the accumulation of metabolic hormones in the hypothalamus could be a significant factor in explaining the link between childhood obesity and central precocious puberty. Additionally, persistent hyperglycemia and insulin resistance in individuals with obesity have been shown to play a role in the function of the HPG axis [18]. Our study also suggests that the fasting insulin levels and insulin resistance index are higher in the adolescent development group compared to the adolescent-undeveloped group. Hyperinsulinemia can lead to a decrease in the levels of sex hormone-binding globulin (SHBG), thereby increasing the bioavailability of sex hormones and stimulating the secretion of androgens in the adrenal glands and ovaries. Previous studies have indicated that increasing insulin sensitivity can reduce androgen levels in the adrenal glands and ovaries and may delay the progression of puberty [19]. In addition, epigenetic changes, such as polymorphisms in the *MKRN3* and *LIN28B* genes, as well as prenatal and postnatal environmental factors, intestinal microbiota, and endocrine disruptors, are also associated with the adolescent development of children and adolescents with obesity [20]. With the discovery of gut microbiota typing and function in recent years, the field of global nutrition has undergone a revolutionary paradigm shift. Changes in the gut microbiota may lead to the production of different metabolites, such as stimulating insulin secretion and the release of peptide hormones that control appetite, thereby contributing to obesity.

Genetic predispositions, climate, diet, nutritional status, socioeconomic factors, physical activity, and health status all impact the development of adolescence, and these same factors also contribute to the development of obesity. In contemporary society, which is rich in material resources, children and adolescents face multiple pressures from academics, parental expectations, and social interactions. These pressures can disrupt cognitive functions such as executive function and self-regulation, and are often alleviated through behaviors such as overeating, consuming high-calorie foods, increasing sedentary activities like watching online videos, reducing physical activity, and shortening sleep duration. Moreover, the widespread availability of fast food in the modern era makes it nearly inevitable that there will be a mismatch between calorie intake and energy expenditure, leading to fat accumulation. This study demonstrates that compared to boys with obesity who have not yet entered adolescence, those in the adolescent development group engage in less exercise and consume more sugary drinks and fried foods. In the case of girls with obesity, the adolescent development group also reports higher daily sitting times and greater intake of sugary drinks and fried foods than the adolescent-undeveloped group. Excessive consumption of fried foods is associated with the development of adolescence in children with obesity. Research from an Italian team concluded that the rising incidence of central precocious puberty may be related to a significant increase in BMI, the increased use of electronic products, and the growing psychological stress [21]. A meta-analysis by Nguyen et al. indicates that a diet high in fiber and monounsaturated fatty acids is associated with later menarche, while a diet high in animal protein and polyunsaturated fats is associated with earlier menarche. The habitual consumption of sugary soft drinks is positively correlated with the risk of early adolescence [22]. A meta-analysis revealed that a diet rich in fiber and monounsaturated fatty acids is associated with later menarche, while a diet high in animal protein and polyunsaturated fats is linked to earlier menarche. Additionally, the regular consumption of sugary soft drinks is positively correlated with the risk of early adolescence [23]. While the pivotal role of nutritional status in the activation of adolescence has been substantiated by numerous studies, the neuroendocrine mechanisms underlying this relationship remain enigmatic. The potential mechanisms by which a high-fat diet may promote adolescent development could include the following: activation of gonadotropin-releasing hormone (GnRH) through hypothalamic microglia; the interplay of intestinal microflora and hormonal actions; or the overexpression of the p53 gene via the Lin28/let-7 system [24].

This study has several limitations: (1) The subjects were regionally restricted, and the sample size was small, which made it impossible to compare sexual development with normal-weight children of the same age and sex. (2) The content of the questionnaire in this study was limited, and there may have been some memory bias when filling it out. However, the questionnaire was designed based on a large-sample epidemiological survey of children’s chronic diseases in China, and its reliability is ensured. (3) The confounding factors controlled in this study were limited, and the research results needed to be verified by high-quality prospective cohort studies. (4) The cross-sectional nature of this study may not be able to definitively prove causality, and geographical factors may limit the broader implementation of some measures. Despite these limitations, our research provides some reliable cross-sectional data on the development of adolescence in 6–12-year-old children with obesity. In the follow-up of this study, it is necessary to further expand the scope of investigation, increase the sample size, continue with longitudinal or interventional studies to track how dietary adjustments or weight management can alter the onset and progression of puberty, which in the hope of obtaining more research conclusions that are of practical clinical significance.

## 5. Conclusions

In conclusion, a high body mass index (BMI) in girls with obesity and high intake of fried foods in both genders are strong predictors of early puberty in school-age children. The interplay of genetics, epigenetics, metabolism, nutrition, and hormones influences the onset and progression of puberty. In childhood obesity, the multiple effects of adipokines (fat factors) have significant impacts on children’s growth and pubertal development. The factors closely related to pubertal development in children with obesity identified through this study may provide a new theoretical basis for preventing early puberty in children with obesity, which is crucial for developing strategies to prevent precocious puberty in children with obesity. Preventing precocious puberty in children with obesity has far-reaching significance for children’s physical and mental health, quality of life, family and societal burden, educational outcomes, and long-term health. However, further research is needed to determine how these new theories can be applied in this field, to elucidate the mechanisms of these factors. Nevertheless, we can still take some practical measures to mitigate the impact of early puberty on the physical and mental health of children with obesity. These measures include controlling the intake of fried foods, increasing the duration of moderate-intensity exercise, and involving joint supervision by parents and schools. Pediatricians provide free dietary counseling and regularly monitor body composition indicators. Such collaborative efforts can more effectively prevent the occurrence of childhood obesity.

## Figures and Tables

**Table 1:** Comparison between the adolescent-undeveloped and adolescent-developed groups of school-age children with obesity.

Factors	Male				Female			
	Puberty Undeveloped (*n* = 83)	Puberty Development (*n* = 69)	Statistic	*p*	Puberty Undeveloped (*n* = 30)	Puberty Development (*n* = 89)	Statistic	*p*
Age (year)	9 (7.81,9.88)	10.92 (9.5,12)	−6.029	<0.001	7.66 (6.94,8.46)	9.25 (8,10.2)	−4.580	<0.001
Weight (Kg)	50.95 (41.85,58.8)	64.7 (53.2,73.1)	−5.732	<0.001	36.55 (32.5,44.3)	48.4 (39.5,60)	−4.079	<0.001
Height (cm)	141.63 ± 10.57	154.29 ± 9.28	−7.765	<0.001	132.42 ± 9.37	143.92 ± 11.68	−5.445	<0.001
BMI	24.4 (22.48,27.07)	26.3 (24.1,29)	−3.109	0.002	21.77 (20.26,23.78)	23.3 (20.9,25.86)	−2.111	0.035
BMI z-score	−0.11 (−0.61,0.58)	0.38 (−0.19,1.09)	−3.109	0.002	−0.79 (−1.18,−0.27)	−0.39 (−1.02,0.27)	−2.111	0.035
Body fat (Kg)	19.74 (15.25,25.18)	23.65 (19.4,29.13)	−3.034	0.002	12.5 (11.2,15.28)	17.39 (13.15,21.4)	−2.979	0.003
Body fat index (kg/m^2^)	9.6 (7.69,12)	10.1 (8.21,11.78)	−0.731	0.465	7.56 (6.6,8.74)	8.21 (6.62,9.91)	−1.195	0.232
Muscle mass (Kg)	29.2 (25.17,32.52)	35.65 (32.5,42.1)	−6.682	<0.001	23 (20.9,26.5)	28.55 (23.97,34.6)	−3.900	<0.001
Muscle mass index (kg/m^2^)	14.4 (13.58,15.03)	15.13 (14.59,16.32)	−4.733	<0.001	13.44 (13.09,14.14)	13.85 (13.25,15.38)	−1.948	0.051
Fat-free weight (Kg)	31 (26.5,34.52)	38.25 (35,44.97)	−6.930	<0.001	24.5 (22.2,28)	30.4 (25.55,36.77)	−3.945	<0.001
Fat-free weight index(kg/m^2^)	15.22 (14.42,15.92)	16.13 (15.54,17.35)	−5.360	<0.001	14.23 (13.81,14.91)	14.72 (14.07,16.33)	−2.139	0.032
Body fat percentage (%)	38.65 (34.15,42.9)	38.3 (33.98,41.3)	0.500	0.617	34.4 (31.8,37.2)	35.2 (31.63,39.75)	−0.602	0.547
Waist/hip ratio	0.84 (0.79,0.89)	0.86 (0.82,0.9)	−2.102	0.036	0.77 (0.76,0.83)	0.82 (0.78,0.87)	−2.346	0.019
Fasting insulin (uU/mL)	17.1 (12.35,27.4)	23.5 (14,33.7)	−2.309	0.021	13.55 (10.07,21.12)	18.7 (12.2,29.9)	−2.370	0.018
Fasting blood sugar (mmol/L)	5.03 ± 0.33	5 ± 0.47	0.435	0.664	4.8 (4.5,5.1)	5 (4.8,5.3)	−2.632	0.008
HOMA-IR	3.9 (2.69,5.89)	5.49 (3.31,7.6)	−2.261	0.024	2.96 (2.2,4.65)	4.24 (2.77,6.93)	−2.594	0.009

**Table 2:** Multiple regression analysis of adolescent development-related factors of children aged 6–12 with obesity.

	Factors	Male					Female				
		B	SE	z	*p*	OR[95%CI]	B	SE	z	*p*	OR[95%CI]
Puberty development	constant	−19.097	4.361	−4.379	<0.001		−9.457	5.719	−1.654	0.098	
	Age	0.150	0.169	0.885	0.376	1.2[0.8,1.6]	0.465	0.290	1.602	0.109	1.6[0.9,2.8]
	Height	0.133	0.038	3.523	<0.001	1.1[1.1,1.2]	0.091	0.046	1.964	0.050	1.1[1,1.2]
	BMI	−0.060	0.090	−0.674	0.5	0.9[0.8,1.1]	0.055	0.024	2.319	0.021	1.06[0.7,1.2]
	Muscle mass index	0.392	0.219	1.787	0.074	1.5[1,2.3]	0.110	0.408	0.269	0.788	1.1[0.5,2.5]
	Waist/hip ratio	−7.639	4.119	−1.855	0.064	0[0,1.5]	−6.968	5.906	−1.180	0.238	0[0,100.2]
	Fasting insulin	−0.002	0.013	−0.121	0.903	1[1,1]	−0.018	0.017	−1.085	0.278	1[0.9,1]

**Table 3:** Comparison of dietary and lifestyle behaviors between the adolescent-undeveloped group and the adolescent-developing group among children with obesity.

Factors	Male			Female		
	Puberty Undeveloped (*n* = 83)	Puberty Development (*n* = 69)	*p*	Puberty Undeveloped (*n* = 30)	Puberty Development (*n* = 89)	*p*
Night sleep time (h)	9 (8.5,9.2)	9 (8.5,9.2)	0.613	9 (8.5,9.4)	8.8 (8.3,9)	0.462
Amount of exercise			0.003			0.82
Less	53 (63.9%)	59 (85.5%)		23 (76.7%)	70 (78.7%)	
More	30 (36.1%)	10 (14.5%)		7 (23.3%)	19 (21.3%)	
Sit-in time (min)	60 (30,120)	60 (40,120)	0.22	30 (20,65)	60 (40,120)	0.003
Meat intake			0.575			0.881
Less	23 (27.7%)	22 (31.9%)		11 (36.7%)	34 (38.2%)	
More	60 (72.3%)	47 (68.1%)		19 (63.3%)	55 (61.8%)	
Sugary beverage intake			<0.001			0.001
Less	72 (86.7%)	36 (52.2%)		26 (86.7%)	46 (51.7%)	
More	11 (13.3%)	33 (47.8%)		4 (13.3%)	43 (48.3%)	
Fried food intake			<0.001			<0.001
Less	80 (96.4%)	36 (52.2%)		28 (93.3%)	40 (44.9%)	
More	3 (3.6%)	33 (47.8%)		2 (6.7%)	49 (55.1%)	
Birth weight (g)	3408 ± 507.5	3574.1 ± 509.9	0.048	3454.8 ± 529.5	3442.8 ± 455.2	0.907
Father’s BMI	24.98 ± 3.35	25.02 ± 2.78	0.063	26.55 ± 2.08	25.67 ± 3.23	0.369
Mother’s BMI	22.20 ± 4.05	22.42 ± 3.53	0.525	23.69 ± 3.87	24.32 ± 4.06	0.806

**Table 4:** Multiple Regression analysis of adolescent development, lifestyle behaviors, and diet among boys.

	Factors	B	SE	z	*p*	OR[95%CI]
Puberty development	constant	−4.662	1.709	−2.728	0.006	
	Amount of exercise	−0.040	0.876	−0.046	0.964	1[0.2,5.4]
	Sugary beverage intake	0.267	0.579	0.461	0.645	1.3[0.4,4.1]
	Fried food intake	2.981	0.765	3.898	<0.001	19.7[4.4,88.2]
	Birth weight	0.000	0.000	0.903	0.367	1[1,1]

**Table 5:** Multiple regression analysis of adolescent development, lifestyle behaviors, and diet among girls.

	Factors	B	SE	z	*p*	OR[95%CI]
Puberty development	constant	−2.914	1.000	−2.914	0.004	
	Sit-in time	0.008	0.005	1.656	0.098	1[1,1]
	Sugary beverage intake	0.229	0.733	0.312	0.755	1.3[0.3,5.3]
	Fried food intake	2.535	0.891	2.845	0.004	12.6[2.2,72.3]

## Data Availability

The datasets presented in this article are not readily available because of privacy considerations. Requests to access the datasets should be directed to zjdct@163.com.
